# Adsorption of Bisphenol A onto β-Cyclodextrin–Based Nanosponges and Innovative Supercritical Green Regeneration of the Sustainable Adsorbent

**DOI:** 10.3390/polym17070856

**Published:** 2025-03-23

**Authors:** Uğur Salgın, İsmail Alomari, Nagihan Soyer, Sema Salgın

**Affiliations:** 1 Department of Chemical Engineering, Faculty of Engineering, Sivas Cumhuriyet University, 58140 Sivas, Turkeyssalgin@cumhuriyet.edu.tr (S.S.); 2 Abu Dhabi National Oil Company, Abu Dhabi P.O. Box 303, United Arab Emirates; smail0949648357@gmail.com

**Keywords:** Bisphenol A, β-Cyclodextrin nanosponges, adsorption, supercritical CO_2_-based green regeneration, sustainable adsorbent materials

## Abstract

Bisphenol A is a widely recognized endocrine disruptor that persists in ecosystems, harms aquatic organisms, and contributes to ecological degradation, raising global environmental concerns. Numerous studies have explored β-cyclodextrin–based adsorbents for Bisphenol A removal; however, their regeneration remains a major challenge, often relying on energy-intensive processes and excessive use of organic solvents. In this study, Bisphenol A was selected as a model pollutant, and its adsorption onto β-cyclodextrin nanosponges was investigated. After adsorption, Bisphenol A was efficiently recovered from the saturated β-cyclodextrin nanosponges using an innovative and sustainable supercritical CO_2_-based green process, which simultaneously regenerated the adsorbent. The adsorption process achieved an efficiency of 95.51 ± 0.82% under optimized conditions (C_0_ = 150 mg/L, m_β-CDNS_ = 0.15 g, T = 25 °C, and N = 200 rpm), with a maximum adsorption capacity of 47.75 ± 0.28 mg/g. The regeneration process achieved over 99% efficiency at 60 °C and 300 bar, with 10% (*v*/*v*) ethanol as a co-solvent, nearly fully restoring the adsorbent’s performance. Unlike conventional regeneration techniques, this green approach eliminates the need for environmentally harmful organic solvents while preserving the adsorbent’s structural integrity, making it a highly efficient and sustainable alternative. This study is the first to demonstrate the effective application of supercritical CO_2_-based regeneration for β-cyclodextrin nanosponges in Bisphenol A removal, providing a scalable and environmentally sustainable solution for wastewater treatment. Furthermore, characterization analyses confirmed that the adsorbent retained its chemical and morphological stability after adsorption and regeneration.

## 1. Introduction

Urbanization, industrialization, and environmental pollution have led to the progressive deterioration of water quality, resulting in the widespread presence of contaminants in aquatic ecosystems [1,2]. Among these contaminants, endocrine-disrupting compounds are of particular concern, as they are exogenous substances capable of modulating endogenous hormonal signaling by either mimicking or inhibiting natural hormone activity, thereby disrupting reproductive and developmental processes in living organisms [3,4,5]. One such endocrine-disrupting compound, Bisphenol A (BPA), has raised significant concerns due to its environmental persistence and adverse effects on human health [4,5,6,7,8,9,10,11]. BPA is a high-production-volume chemical predominantly used as a monomer in the synthesis of polycarbonate plastics and epoxy resins, accounting for most of its global production [12,13]. These synthetic polymers are extensively employed across a wide range of consumer and end-use industrial sectors, including packaging, healthcare and medical devices, electronics, automotive manufacturing, construction materials, and the maritime and defense industries, as well as paper, printing, textiles, and polymer additive applications [5]. Due to its widespread use and environmental persistence, BPA remains one of the most prevalent industrial chemicals worldwide [14]. The global BPA market is projected to grow from 8.72 million tons in 2025 to 11.98 million tons by 2030, with an estimated compound annual growth rate of 6.56% [15]. However, inadequate waste management and inefficient wastewater treatment systems may further exacerbate BPA contamination of surface and groundwater, posing substantial risks to both environmental and human health.

In response to these concerns, regulatory agencies have established concentration limits for BPA in various water sources, although these limits vary significantly across regions. For instance, China enforces a 0.1 mg/L limit for industrial wastewater [16], while Canada sets a stricter 3.5 µg/L threshold for surface water [17]. The European Union regulates BPA in drinking water at 2.5 µg/L, with proposed environmental quality standards of 17.4 ng/L for freshwater and 0.034 ng/L for food to protect human health [18]. France applies an even stricter 0.1 µg/L limit, whereas Germany allows up to 30 µg/L [19]. In the United States, no official BPA limit exists, but surface water levels are typically below 1 µg/L [20]. To safeguard aquatic ecosystems, BPA concentrations should ideally remain within 1–15 µg/L in various water sources. Additionally, international drinking water standards further regulate BPA levels to mitigate its potential risks [19].

Industrial wastewater from BPA-intensive sectors can exhibit significantly higher concentrations before undergoing treatment. For example, BPA levels can reach approximately 300 mg/L in polycarbonate production [21], 50 mg/L in epoxy resin manufacturing [22], and up to 33.5 mg/L in wastepaper recycling [23]. As a result, significant research efforts have been directed toward developing efficient removal strategies for BPA-contaminated water. Various treatment methods have been explored, including physical (adsorption, membrane separation, liquid–liquid extraction), chemical (advanced oxidation processes, ozonation, electrochemical oxidation), and biological (microbial, enzymatic, algal-based) techniques [13,24]. Advanced oxidation processes such as ozonation, Fenton and photo-Fenton reactions, photocatalysis using titanium dioxide, and ultrasonic cavitation rely on highly reactive hydroxyl radicals to degrade BPA, but challenges such as optimization issues, potential toxic by-product formation, and high operational costs hinder large-scale applications [25,26,27]. Membrane filtration techniques remove BPA via size exclusion and charge-based interactions, but membrane fouling, energy consumption, and pre-treatment requirements limit their widespread industrial use [6,28,29,30,31,32]. Liquid–liquid extraction, another physical separation technique, exploits BPA’s solubility in immiscible solvents but is constrained by high solvent usage, chemical consumption, and secondary waste generation. Biological treatments, including microbial and enzymatic degradation, offer an environmentally friendly solution but are relatively slow and sensitive to environmental fluctuations, while membrane bioreactors improve biodegradation efficiency but face limitations such as bacterial loss and incomplete mineralization [33,34,35]. Among these approaches, adsorption has emerged as one of the most effective, cost-efficient, and scalable methods [36]. While advanced oxidation processes and membrane filtration can achieve removal rates between 70% and 100%, and biological processes range from 20% to 100%, adsorption typically reaches 80–100% efficiency [37]. Unlike membrane technologies, which require extensive maintenance, or advanced oxidation processes, which are energy-intensive, adsorption offers a simple, low-cost, and environmentally sustainable alternative, making it a practical choice for large-scale BPA removal [13,19,36,37,38].

The removal of BPA from aqueous environments through adsorption-based methods has been extensively studied due to their cost-effectiveness, high adsorption efficiency, and ease of operation. The adsorption mechanism of BPA involves electrostatic forces, π–π interactions, hydrophobic interactions, acid–base interactions, hydrogen bonding, and pore filling, allowing different adsorbent materials to capture BPA effectively. A wide range of adsorbents has been explored for BPA removal, including carbon-based materials, porous frameworks, polymer-based adsorbents, and mineral-based adsorbents [13,24]. Carbon-based adsorbents, such as activated carbon [7], carbon nanotubes [39], graphene derivatives [40], and biochar [41], exhibit high adsorption capacities and adsorption efficiencies of 80–99% due to their large surface areas, high porosity, and strong π–π interactions. However, high production costs and the need for frequent regeneration can limit their large-scale application. Porous materials, including metal-organic frameworks [42] and covalent organic frameworks [34], offer tunable adsorption properties, selective binding sites, and excellent structural stability, achieving adsorption efficiencies of 85–99%. Clays [43] and zeolites [44], while cost-effective alternatives, generally exhibit lower adsorption capacities and adsorption efficiencies (60–90%) due to their weaker surface interactions. Polymer-based adsorbents, including chitosan [45], cyclodextrin (CD) polymers, and molecularly imprinted polymers [46], offer selective binding sites, enhancing their efficiency in BPA removal [13,24]. Among these, β-CD–based polymers are particularly noteworthy due to their ability to form host–guest inclusion complexes, enabling strong molecular interactions with BPA while maintaining excellent reusability and modification potential [8,47,48]. A hyper-crosslinked β-CD porous polymer synthesized via the Friedel–Crafts reaction with dichloroxylene and benzylated β-CD demonstrated high adsorption capacity, an adsorption efficiency of 88%, and outstanding thermal stability, making it a viable candidate for industrial applications [8]. Additionally, a β-CD–based polyurethane synthesized via crosslinking with 4,4′-diphenylmethane diisocyanate exhibited an exceptionally high specific surface area and remarkable adsorption capacity for BPA. Even after six adsorption–desorption cycles, it maintained an adsorption efficiency above 90%, highlighting its stability and reusability [48]. A novel hyper-crosslinked polymeric adsorbent modified with acetyl aniline as a crosslinking bridge demonstrated higher adsorption capacity than commercial resins, achieving near 100% regeneration efficiency using ethanol and sodium hydroxide treatment, indicating excellent long-term performance [49].

CDs are cyclic oligosaccharides with a toroidal structure, characterized by a hydrophilic outer surface and a hydrophobic internal cavity, which allows them to encapsulate hydrophobic pollutants, such as BPA, via host–guest interactions. CDs are classified into α-, β-, and γ-CDs based on their cavity size, with β-CD being the most widely used due to its optimal complexation ability, stable structure, low cost, and availability [50,51]. However, the limited solubility of β-CD requires chemical modification or crosslinking with epichlorohydrin (EPI) or other aliphatic and aromatic crosslinking agents to form an insoluble network, thus enhancing the structural stability, rigidity, and adsorption efficiency of the resulting material while facilitating the formation of micro-mesoporous structures, thereby improving its reusability for environmental applications [52,53,54].

The increasing interest in β-CDNSs for wastewater treatment stems from their exceptional adsorption properties. However, current adsorption processes face sustainability challenges, necessitating the development of cyclical and eco-friendly regeneration strategies. Traditional thermal or solvent-based regeneration methods often pose environmental risks and increase secondary waste production. In contrast, supercritical CO_2_ (scCO_2_)-based regeneration offers a promising green alternative, leveraging its superior diffusion and solvating power to enhance pollutant desorption while minimizing hazardous chemical usage. Recent studies [55,56] have demonstrated the effectiveness of scCO_2_ for adsorbent recovery and pollutant removal, yet its inherent limitations—particularly its nonpolar nature—require further investigation to improve its applicability for the desorption of polar contaminants like BPA. While BPA adsorption onto β-CDNSs has been explored in the literature, to the best of our knowledge, there have been no studies investigating scCO_2_-based green regeneration for β-CDNSs, highlighting the novelty of this research as a significant contribution to this area. This study aimed to address this gap by optimizing scCO_2_ regeneration efficiency through the strategic incorporation of co-solvents, thereby enhancing pollutant desorption while maintaining the stability and reusability of β-CDNSs. A key challenge in scCO_2_-based regeneration is its limited interaction with polar pollutants due to its lack of polarity, which affects desorption efficiency. To address this, the incorporation of co-solvents or entrainers, such as ethanol, enhances the solvating power of scCO_2_ by modifying phase behavior and increasing its affinity for polar solutes [55,56,57]. The addition of entrainers influences mass transfer parameters, including diffusivity and viscosity, facilitating better penetration into porous structures and improving desorption kinetics [57]. Furthermore, entrainers alter thermodynamic equilibrium by enhancing intermolecular interactions, leading to increased BPA solubility in scCO_2_ and more efficient pollutant removal. While previous studies have investigated the adsorption and desorption of organic pollutants using β-CDNSs, they have primarily focused on conventional thermal and solvent-based regeneration techniques, which suffer from inefficiency and environmental concerns. This research pioneers the application of scCO_2_-based green regeneration for β-CDNSs, introducing entrainer-enhanced desorption to improve efficiency, a method that has not been comprehensively explored in the context of BPA removal.

This research systematically investigates the process of BPA adsorption onto β-CDNSs, evaluating main adsorption parameters such as initial BPA concentration, β-CDNS dosage, shaking speed, and adsorption temperature. To improve accuracy, Python-based computational methods are employed for nonlinear regression and numerical optimization to determine adsorption isotherm (12 models) and adsorption kinetic (6 models) model parameters. Additionally, thermodynamic feasibility is evaluated through spontaneity and enthalpy-entropy analyses. By optimizing key regeneration process factors such as temperature, pressure, and entrainer concentration, this study aimed to establish a controlled and scalable strategy for enhancing scCO_2_-based pollutant regeneration. To enhance sustainability, an scCO_2_-based green regeneration strategy was explored as an environmentally friendly alternative to conventional desorption methods, aiming to extend β-CDNS usability while minimizing secondary waste generation. Furthermore, structural and morphological stability during adsorption and regeneration was analyzed using scanning electron microscopy (SEM), Fourier transform infrared spectroscopy (FTIR), and differential scanning calorimetry (DSC). By integrating computational modeling, experimental adsorption studies, and sustainable regeneration techniques, this study makes a significant contribution to the field as the first to explore scCO_2_-based green regeneration for β-CDNSs, demonstrating its feasibility and industrial applicability for BPA removal.

## 2. Materials and Methods

### 2.1. Materials

β-CD (≥97%) and the crosslinking agent EPI (≥99%) were obtained from Sigma-Aldrich Chemie GmbH (Taufkirchen, Germany) for the synthesis of β-CDNP. BPA (99%, Acros Organics, Geel, Belgium), which was employed as the organic contaminant, was procured from ThermoFisher GmbH (Kandel, Germany). Organic solvents, such as acetone (≥9799.5%) and ethanol (≥99.8%), were acquired from Sigma-Aldrich Chemie GmbH (Taufkirchen, Germany). NaOH, which was utilized for preparing the base solution, was sourced from Merck KGaA (Darmstadt, Germany). Deionized water with a conductivity of 18.2 MΩ cm, produced using the Milli-Q^®^ Gradient Model purification system (Millipore Co., Billerica, MA, USA), was used to prepare the BPA solutions. Liquid CO_2_ (≥99.995%), which was applied for desorbing BPA from β-CDNS in a supercritical green regeneration, was supplied by Günaylar Industrial Medical Gas Production and Filling Facilities (Istanbul, Türkiye).

### 2.2. Synthesis of β-Cyclodextrin Nanosponges

The β-CDNSs were synthesized using a modified method based on Salgın et al. (2016) [58]. In this process, β-CD was used at a 50 *w*/*v* ratio in 100 mL of a 40% wt. NaOH solution. To obtain a homogeneous mixture, the solution was stirred at 200 rpm for 30 min using a magnetic stirrer equipped with temperature and stirring speed control (Stuart™ UC152D Hot Plate Stirrer + STC1 Temperature Controller Model, BibbyScientific Ltd., Staffordshire, UK). Before adding the crosslinking agent EPI, the temperature of the synthesis medium was increased to 55 °C and maintained at this temperature throughout the process. The crosslinking reaction between β-CD and EPI was performed by adding EPI dropwise over 1 h at 200 rpm and 55 °C until the final volume reached 190 mL. The reaction then continued for an additional 30 min under the same conditions. Upon completion of the reaction, unreacted substances, particularly β-CD and EPI, were removed through a series of washing steps. The washing process was initially performed until the pH of the medium reached 7, with the pH monitored during each step using a Sartorius PB-11 pH meter (Sartorius AG, Göttingen, Germany). The filtered β-CDNSs were transferred to a cellulose cartridge (Whatman^®^ Extraction Thimbles, Whatman International, Ltd., Maidstone, UK) and washed with 250 mL of acetone using a Soxhlet extraction unit for 12 h. Following this process, drying and grinding steps were performed. The β-CDNSs were dried in a vacuum oven at 25 °C for 1 day. The dried material was ground using an agate mortar and sieved to obtain particles of different sizes. For adsorption experiments, β-CDNSs within a 150–500 µm size range were used.

### 2.3. Batch Adsorption Experiments

Model wastewater was prepared by dissolving different concentrations of BPA in deionized water at the natural pH of the solutions, and synthesized β-CDNSs were used as adsorbents. Batch adsorption of BPA onto β-CDNSs was tested using a temperature- and shaking speed–controlled orbital shaker (Barnstead International/LAB Line MAXQ 4000 Model, Melrose Park, IL, USA). The experiments were performed in airtight amber glass bottles (V = 100 mL total volume), each containing V = 50 mL of solution with predefined concentrations of the adsorbent and β-CDNS. The adsorption performance of the β-CDNSs was evaluated under various operating conditions, including contact time (up to 18 h), adsorbent dosage (m_β-CDNS_ = 0.075–0.45 g), initial BPA concentration (C_0_ = 5–300 mg/L), and operating temperature (T = 15–45 °C). The selected BPA concentration range aligns with previous studies that have investigated adsorption performance at varying levels, often extending to high concentrations. For instance, Du and Piao (2018) examined BPA adsorption on hydrogel microparticles using an initial BPA concentration range of 25–1000 mg/L [59]. Wang and Zhang (2020) studied BPA adsorption on biochar and magnetic biochar with initial concentrations ranging from 8 to 200 mg/L [60]. Rovani et al. (2020) conducted adsorption experiments on mesoporous silica nanoparticles using 100 mg/L of BPA [61]. Martín-Lara et al. (2020) tested BPA adsorption at concentrations between 1 and 160 mg/L [62]. Although BPA is often detected at trace levels in surface and drinking water (e.g., 12 µg/L in stream water and 3.5–59.8 ng/L in drinking water), industrial wastewater from BPA-intensive sectors can exhibit significantly higher concentrations, sometimes exceeding 100 mg/L [62,63]. Thus, our chosen range (5–300 mg/L) reflects both environmentally relevant and industrially significant conditions, ensuring a comprehensive evaluation of adsorption efficiency, kinetics, and isotherm behavior. Each data point in the figures represents the average of three independent adsorption experiments, with the standard deviation (expressed as ±).

The BPA concentration in the liquid phase was determined using a UV-visible spectrophotometer (Model 1800, Shimadzu Inc., Kyoto, Japan) at a maximum wavelength of 276 nm. The molar absorptivity was determined from the slope of the calibration curve as 0.0138 L/mg cm, with an R^2^ value of 0.9998. The adsorption efficiency (E%) and the adsorption capacity (q) for any contact time were calculated using the following equations:

(1) $$E\%=\frac{C_{0}-C_{t}}{C_{0}}\times 100$$

(2) $$q=\frac{C_{0}-C_{t}}{m_{\beta -CDNS}}\times V$$

where C_0_ and C_t_ are the initial and time-dependent BPA concentrations (mg/L), respectively, with t expressed in minutes; V is the volume of the BPA solution (L); and m_β-CDNS_ is the mass of the adsorbent used (g).

### 2.4. Regeneration Experiments Using Supercritical CO_2_-Based Green Solvent

**Figure 1:**
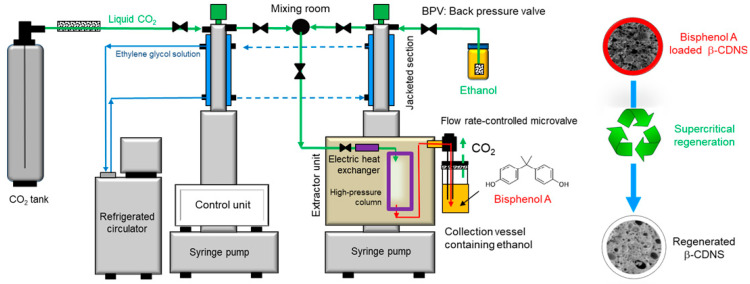
Process flow diagram of the regeneration system.

The regeneration process of β-CDNS saturated with BPA was conducted in a supercritical fluid extraction system (ISCO SFX 220 Model, Isco Inc., Lincoln, NE, USA), operating continuously in the liquid phase and in batch mode in the solid phase, with controlled parameters such as temperature, pressure, CO_2_ and co-solvent flow rates. The process flow diagram is shown in Figure 1.

Approximately 0.45 g of BPA-saturated adsorbent was placed in a high-pressure column with an internal volume of 10 mL. Filters with a porosity of 0.5 µm were installed at the inlet and outlet of the high-pressure column. Liquid CO_2_ from the tank was pressurized to the desired regeneration pressure using a high-pressure syringe pump and subsequently fed into the unit containing the high-pressure column. The CO_2_ was cooled to 4 °C through the jacketed section of the pump head using an ethylene glycol solution supplied by a refrigerated circulator (Haake C25P Phoenix II, ThermoElectron Co., Waltham, MA, USA). During the regeneration process, the CO_2_ pressurized to the desired operating pressure was passed through an electric heat exchanger within the extractor unit containing the high-pressure column to ensure it reached the desired regeneration temperature. BPA desorbed by the SCF was collected in a product collection vessel containing ethanol after passing through a temperature- and flow rate-controlled microvalve set to 70 °C and maintained at Q = 2 ± 0.2 mL/min. In experiments investigating the effect of ethanol concentration as a co-solvent (entrainer), a second high-pressure syringe pump was used to deliver the CO_2_ + ethanol mixture at the desired ratio into the high-pressure column. The regeneration process was performed at a constant flow rate, under varying regeneration pressures (200 and 300 bar), regeneration temperatures (60 and 80 °C), and co-solvent concentrations (5 and 10% (*v*/*v*)), for a duration of 3 h, with all processes monitored and controlled through the control unit. Each data point in the figures represents the average of three independent regeneration experiments, with the standard deviation (expressed as ±).

The BPA concentration in the ethanol phase was determined using a UV-visible spectrophotometer (Model 1800, Shimadzu Inc., Kyoto, Japan) at a maximum wavelength of 279 nm. The molar absorptivity was determined from the slope of the calibration curve as 0.0165 L/mg cm with an R^2^ value of 0.9984. The regeneration yield (R%) was calculated using the following equation:

(3) $$R\%=\frac{m_{0}-m_{t}}{m_{0}}\times 100$$

where m_0_ and m_t_ represent the mass (mg) of BPA loaded onto the adsorbent and the mass (mg) of BPA removed from the adsorbent at the end of the regeneration process.

### 2.5. Characterizations of β-Cyclodextrin Nanosponges

Structural characterization of β-CDNSs before and after adsorption and scCO_2_-based green regeneration processes was performed using an FTIR spectroscope (Spectrum 100 Model, Perkin Elmer, Inc., Norwalk, CT, USA) equipped with a diamond ATR (Attenuated Total Reflectance) unit. The FTIR spectra were recorded at room temperature with a resolution of 4 cm^−1^ within the spectral range of 400–4000 cm^−1^. The technique was employed to detect characteristic vibrational bands and identify any structural changes in β-CDNSs that occurred during the adsorption of BPA and subsequent regeneration processes.

The thermal behavior of β-CDNSs before and after both the adsorption process and the scCO_2_-based green regeneration process, as well as those of BPA and β-CD, was analyzed using a DSC 7020 instrument (Hitachi High-Tech Science Corporation, Tokyo, Japan) operating in a flowing N_2_ atmosphere with 99.995% purity at a flow rate of 20 mL/min. For the analysis, an 8 mg sample was weighed and placed in hermetically sealed aluminum pans, with a hermetically sealed empty aluminum pan used as a reference. The thermograms were recorded from 20 to 290 °C using a temperature gradient of 10 °C/min. This technique was employed to assess the thermal stability and phase transitions of the materials before and after the adsorption and regeneration processes.

Morphological analysis of β-CDNSs was conducted using a Tescan Mira 3 scanning electron microscope (Tescan Orsay Holding a.s., Brno, Czech Republic) at an acceleration voltage of 10 kV. To minimize surface charging effects, all samples were sputter-coated with a 5 nm gold layer prior to imaging. The SEM images were taken at a magnification of 500×. The built-in software of the scanning electron microscope was used to evaluate porosity, pore size distribution, surface roughness, fractal complexity, and pore shape regularity.

Laser diffraction particle size (LDPS) analysis measurements were performed to control the agglomeration, swelling, and solubility of the β-CDNSs in deionized water as a dispersant medium. The average particle sizes (Dv10, Dv50, Dv90) and particle size distributions of the β-CDNS samples were determined using a Mastersizer 3000 analyzer (Malvern Instruments Ltd., Worcestershire, UK) that was connected to a wet dispersion unit (Hydro MV, Malvern Instruments Ltd., Worcestershire, UK). NSs were added to the dispersion unit until the obscuration value was approximately 10 wt.%. The ultrasonic probe, stirring speed, and temperature were set to 20%, 1500 rpm, and 25 °C, respectively. The refractive and absorption indices of the β-CDNSs were 1.335 and 0.01, respectively. The measurement sequence setting of the software program on the Mastersizer computer allowed us to specify the number of measurements and the duration of any delay between measurements [64].

### 2.6. Computational Approaches for Adsorption Isotherm, Kinetics, and Thermodynamics

Nonlinear regression analysis was applied to determine the parameters of adsorption isotherms and kinetic models and assess their fit to the experimental data. The curve_fit function from the SciPy (version 1.14.1) library in the Python computer program (version 3.11.11, Python Software Foundation, Wilmington, DE, USA) was used to fit nonlinear kinetic models. Since the mixed-order kinetic model lacks a closed-form analytical solution, it was fitted using a numerical optimization approach. The governing differential equation was solved numerically using the odeint function from the SciPy library, which employs the LSODA algorithm. The optimal kinetic parameters were determined by minimizing the residual sum of squares between experimental and predicted values.

The accuracy of the models was evaluated using the coefficient of determination (R^2^), calculated as follows:

(4) $$R^{2}=1-\frac{\sum {\left(q_{exp}-q_{pred}\right)}^{2}}{\sum {\left(q_{exp}-\bar{q}\right)}^{2}}$$

where q_exp_ is the experimental adsorption capacity, q_pred_ is the model-predicted value, and $\bar{q}$ is the mean of the experimental values.

The equilibrium constant (K_C_) was calculated as the ratio of the amount of BPA adsorbed onto β-CDNS at equilibrium to the amount of BPA remaining in the aqueous BPA solution at equilibrium. Equilibrium was confirmed by ensuring that q_e_ and C_e_ remained stable over time. A system is considered to have reached equilibrium when the equilibrium concentration in the solution (C_e_) and the amount of adsorbed substance (q_e_) no longer change over time. Additionally, if measurements taken at different time intervals show no variation in the amount of adsorbed BPA or its concentration in the solution, equilibrium is assumed to be established.

In the thermodynamic analysis, the standard Gibbs free energy change (ΔG^o^) was calculated using the following equation:

(5) $${∆G}^{o}=-RTlnK_{C}$$

where R is the universal gas constant and T is the absolute temperature.

Furthermore, changes in enthalpy (ΔH^o^) and entropy (ΔS°) were estimated using the van’t Hoff equation, where linear regression was employed, as shown in Equation (6):

(6) $$lnK_{C}=\frac{{∆S}^{o}}{R}-\frac{{∆H}^{o}}{RT}$$

Experiments were conducted under the assumption that enthalpy (ΔH°) and entropy (ΔS°) remained constant over the studied temperature range. The van’t Hoff equation (Equation (6)) was applied based on the assumption of a linear relationship, with heat capacity changes considered negligible. These assumptions facilitated the calculation of thermodynamic parameters based on the obtained equilibrium data.

## 3. Results and Discussion

### 3.1. Adsorption Parameters: Effects on Efficiency and Capacity

#### 3.1.1. Effect of Adsorbent Dosage

**Figure 2:**
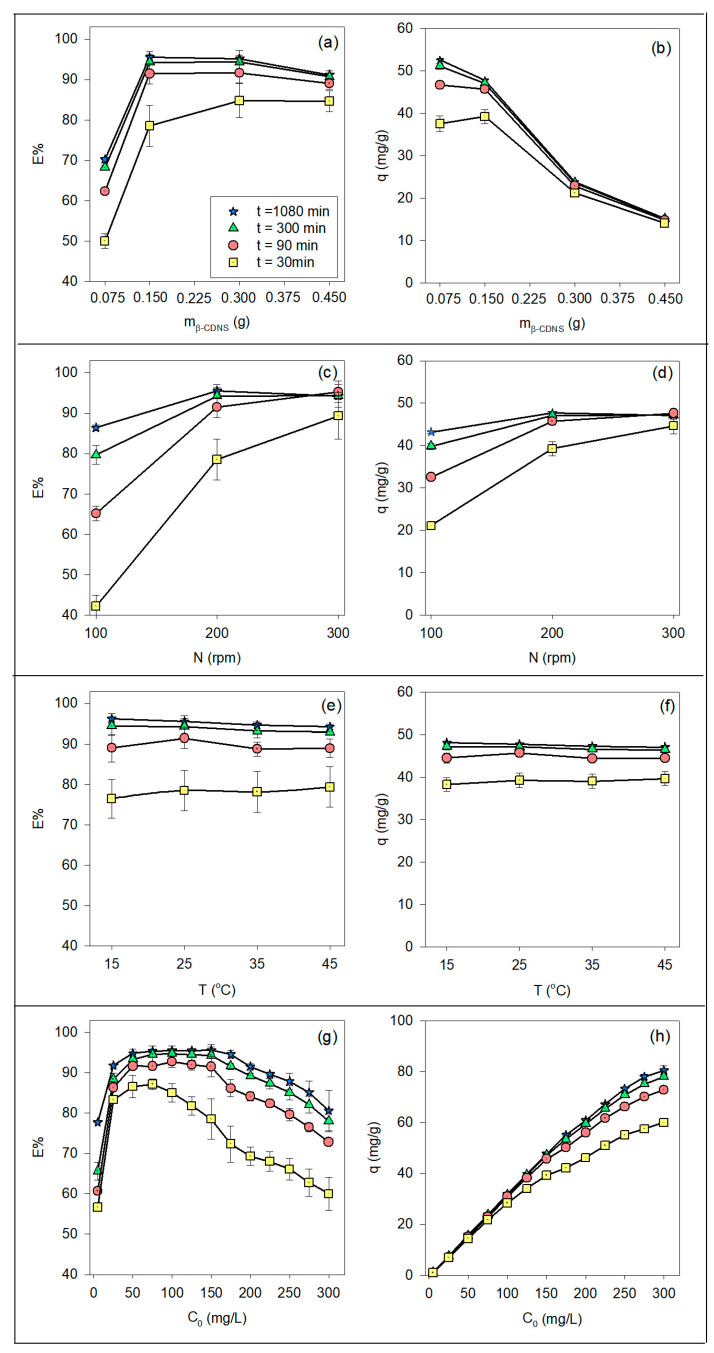
Effects of β-CDNS dosage (**a**,**b**), shaking speed (**c**,**d**), adsorption temperature (**e**,**f**), and initial BPA concentration (**g**,**h**) on the adsorption efficiency (E%) (**a**,**c**,**e**,**g**) and adsorption capacity (q) (**b**,**d**,**f**,**h**) at different contact times.

The effects of β-CSNS dosage (m_β-CDNS_) on BPA adsorption efficiency (E%) and adsorption capacity (q) at different contact times are shown in Figure 2a and Figure 2b, respectively. The experiments were conducted under the following conditions: C_0_ = 150 mg/L, T = 25 °C, and N = 200 rpm. As observed in Figure 2a, E% increased with adsorbent dosage, reaching its maximum value (~95%) at both 0.15 g and 0.30 g after 1080 min. At lower adsorbent dosages (0.075 g), E% was significantly lower (70.22 ± 0.06%), particularly at shorter contact times (30–90 min), but increased substantially with extended adsorption duration. However, beyond 0.30 g, no further improvement was observed, and E% stabilized at 91.18 ± 0.47% at 0.45 g. This stabilization suggests that excessive absorbent caused adsorption site saturation and steric hindrance, resulting in no additional contribution to removal efficiency. These results align with previous studies on activated carbon [62] and biochar [60], where adsorption efficiency plateaued beyond an optimal adsorbent dose. Additionally, Łukasik et al. (2023) and Gong et al. (2020) reported similar saturation trends in modified CD-based adsorbents, where excess functionalized surfaces reached equilibrium, leading to no additional adsorption improvement [2,3]. However, unlike those studies, the β-CDNS adsorbent in this work is an unmodified CD network system, allowing direct insight into the intrinsic host–guest interactions governing adsorption. Studies such as Lv et al. (2021) and Cheng and Wang (2024) suggest that chemical modifications on CD-based adsorbents enhance adsorption efficiency by introducing additional functional groups, yet our results indicate that even unmodified β-CSNS achieves high adsorption efficiency, reinforcing its potential as a sustainable adsorbent material [13,65]. BPA adsorption efficiencies reported in the literature vary from 87% to 95%, depending on the adsorbent type [2,3,13,60,62,65].

Conversely, Figure 2b demonstrates that q decreases with increasing adsorbent dosage across all contact times, indicating inefficient utilization of adsorption sites at higher doses. Specifically, q was highest at 0.075 g, with a value of 52.67 ± 0.06 mg/g, and decreased significantly to 15.20 ± 0.05 mg/g at 0.45 g, suggesting an increase in the number of unused active sites. Beyond 0.30 g, q values converged across all contact times, indicating that steric hindrance and adsorption sites overlap likely occurred, thus reducing the per-unit adsorption efficiency. This trend is consistent with findings from Lv et al. (2021), who reported an inverse correlation between q and adsorbent dosage due to inefficient site utilization [65], and from Gong et al. (2020), who found that, while increasing adsorbent mass improves removal efficiency, per-unit adsorption capacity declines in functionalized CD-based adsorbents [3].

Furthermore, Martín-Lara (2020) and Zang Zhang (2020) observed similar behavior for activated carbon and biochar, where excessive dosages led to solute competition and reduced adsorption efficiency per gram of adsorbent [60,62]. Likewise, Łukasik et al. (2023) emphasized that, even in modified CD-based adsorbents, excessive material can hinder adsorption due to site aggregation, a phenomenon that is also evident in this study with unmodified β-CDNS [2]. These findings reinforce that an adsorbent dosage of ~0.15 g provides the best balance between maximizing E% (95.51 ± 0.83%) and maintaining effective q (47.75 ± 0.28 mg/g), preventing excessive material usage while demonstrating that unmodified β-CDNS performs comparably to modified CD-based systems, highlighting its viability as a cost-effective and environmentally friendly alternative for BPA removal.

#### 3.1.2. Effect of Shaking Speed

The effect of shaking speed (N) on the E% and q of BPA onto β-CSNS at different contact times is shown in Figure 2c,d. The experiments were conducted under the following conditions: C_0_ = 150 mg/L, T = 25 °C, and m_β-CDNS_ = 0.15 g. E% increased with shaking speed, reaching its maximum at 200 rpm (95.51 ± 0.83%), beyond which further increases did not significantly enhance adsorption efficiency, which stabilized around 94.26 ± 0.81%. This trend aligns with the fundamental adsorption mechanism, which involves three main steps: (i) mass transfer from bulk solution to the adsorbent surface, (ii) diffusion through the liquid film surrounding the adsorbent, and (iii) adsorption onto active sites (chemisorption or physisorption) [66]. The influence of shaking speed is particularly critical in steps (i) and (ii), where it enhances mass transfer efficiency and reduces boundary layer resistance.

A similar trend was observed for q, which increased with shaking speed, reaching 47.75 ± 0.28 mg/g at 1080 min at 200 rpm. However, at 300 rpm, a slight decrease (47.13 ± 0.27 mg/g) was observed, suggesting that excessive turbulence disrupted weak adsorbate–adsorbent interactions, causing minor desorption effects. These results were consistent with Gong et al. (2020), who observed no significant improvement in adsorption beyond 150 rpm, indicating a general trend where excessive shaking speed did not enhance performance, possibly due to limitations in mass transfer and interactions within the system [3]. Additionally, shaking speed significantly influenced adsorption kinetics, affecting the rate at which equilibrium was achieved. At 100 rpm, higher mass transfer resistance led to slower adsorption, with E% reaching only 42.22 ± 2.71% at 30 min. In contrast, at 200 rpm, E% rapidly increased to 78.50 ± 5.05% after 30 min, demonstrating improved mass transfer efficiency. Although 300 rpm allowed equilibrium to be reached faster (E% = 95.27 ± 2.66% within 90 min), it did not further increase adsorption capacity, reinforcing the idea that excessive turbulence may hinder stable adsorption by causing desorption of weakly bound molecules. These findings are particularly relevant for practical wastewater treatment applications. While increasing shaking speed can enhance adsorption kinetics, excessive agitation does not always translate to higher adsorption capacity. In large-scale wastewater treatment systems, optimizing shaking speed is crucial for balancing energy efficiency and pollutant removal. Our results suggest that 200 rpm is the optimal balance between enhancing mass transfer and avoiding unnecessary energy expenditure. Thus, our study confirms that, while agitation plays a crucial role in adsorption, excessive turbulence beyond a critical speed (200 rpm) can lead to diminishing returns due to desorption effects and mass transfer limitations. These observations align with previous studies [3,67], which also reported that, beyond an optimal speed (typically 150–200 rpm), further increases in shaking speed do not contribute significantly to adsorption performance.

#### 3.1.3. Effect of Adsorption Temperature

The effects of adsorption temperature (T) on the E% and q of BPA onto β-CSNS at different contact times are shown in Figure 2e and Figure 2f, respectively. The experiments were conducted under the following conditions: C_0_ = 150 mg/L, m_β-CDNS_ = 0.15 g, and N = 200 rpm. E% remained relatively stable at different temperatures. At 15 °C, E% reached 96.23 ± 1.24% at 1080 min, while at 45 °C, it slightly decreased to 94.22 ± 0.62%. Similarly, at 25 °C and 35 °C, adsorption efficiency remained within a close range, at 95.51 ± 0.83% and 94.64 ± 0.84%, respectively. These findings suggest that temperature variations do not significantly affect adsorption efficiency, indicating that the process is not strongly temperature-dependent. q exhibited a similar trend. At 15 °C, q was 48.12 ± 0.41 mg/g at 1080 min, while at 45 °C, it slightly decreased to 47.11 ± 0.21 mg/g. The intermediate temperatures of 25 °C and 35 °C yielded q values of 47.75 ± 0.28 mg/g and 47.32 ± 0.28 mg/g, respectively. These results suggest that adsorption capacity is only marginally influenced by temperature changes, further reinforcing the conclusion that the adsorption process is largely independent of temperature within the studied range.

Comparison with Gong et al. (2020) revealed a notable difference in temperature dependence [3]. While the present study demonstrates that adsorption efficiency remains stable across a broad temperature range (15–45 °C), the findings of Gong et al. (2020) indicate that BPA removal efficiency increases with temperature up to 30 °C but declines at higher temperatures. This suggests that the adsorption mechanism in the study by Gong et al. (2020) was more sensitive to thermal variations, possibly due to differences in adsorbent properties or interaction energies. Furthermore, Gong et al. concluded that adsorption was an exothermic process, with higher temperatures reducing adsorption efficiency, whereas in the present study, temperature effects were minimal, suggesting a different adsorption mechanism or stronger affinity between BPA and the adsorbent.

Similarly, Sirach and Dave (2023) found that higher temperatures led to an increase in desorption, which resulted in a decrease in overall adsorption efficiency, supporting the idea that thermal energy disrupts adsorbate–adsorbent interactions in certain systems [9]. Li et al. (2023) also observed a slight decline in BPA adsorption efficiency at elevated temperatures, likely due to weakened intermolecular forces between the adsorbent and BPA molecules [68]. However, in contrast to these studies, Gupta et al. (2017) reported an endothermic adsorption process in which higher temperatures facilitated greater BPA uptake, suggesting an adsorption mechanism driven by enhanced molecular diffusion and increased active site availability [11]. Compared to these findings, the present study demonstrates a temperature-independent adsorption behavior, in which neither E% nor q exhibited significant variations with changing temperature. This suggests that the adsorbent used in this study may possess highly stable adsorption sites that are less susceptible to thermal fluctuations, possibly due to strong surface interactions or low thermal activation energy requirements.

#### 3.1.4. Effect of Initial Bisphenol A Concentration

The effects of initial BPA concentration (C_0_) and contact time on the E% and q onto β-CDNS are shown in Figure 2g and Figure 2h, respectively. The experiments were conducted under the following conditions: T = 25 °C, m_β-CDNS_ = 0.15 g, N = 200 rpm. The results show that E% increased with C_0_, reaching a maximum of 95.51 ± 0.82% at 150 mg/L after 1080 min. Beyond this concentration, E% declined due to site saturation, decreasing to 80.54 ± 4.97% at 300 mg/L. In contrast, q continued to increase, reaching 80.54 ± 1.66 mg/g at 300 mg/L. This trend suggests that, while higher C_0_ enhanced mass transfer, the limited number of active sites ultimately restricted further improvement in E%. The time-dependent behavior of E% reveals that, at low initial concentrations, adsorption efficiency significantly increased with prolonged contact time, reaching approximately 95% after 1080 min. At higher C_0_, E% stabilizes around 80–85%, indicating site saturation and competitive binding effects. Meanwhile, q exhibits a continuous increase across all conditions, confirming that extended contact time enhances adsorption, particularly at elevated C_0_. The adsorption rates indicate a rapid initial uptake within the first 90 min, which is likely driven by the high availability of active sites. After this phase, the adsorption rate slows as the remaining available sites become occupied. At lower C_0_, equilibrium is reached more quickly, whereas at higher concentrations, adsorption continues gradually over extended contact times. These rates suggest that adsorption follows a multi-stage process, where external diffusion dominates in the early stages, while intraparticle diffusion becomes more significant as equilibrium approaches. These findings align with previous studies on β-cyclodextrin–based adsorbents, where E% reaches its peak at moderate C_0_ before declining due to surface saturation. Similar trends were reported by Gupta et al. (2017) for a graphene oxide–β-cyclodextrin nanocomposite [11]. The results confirm the strong q of β-CDNS for BPA removal, with E% influenced by C_0_ and contact time.

### 3.2. Computational Optimization of Adsorption Models: Isotherm, Kinetics, and Thermodynamics

#### 3.2.1. Adsorption Isotherms

The adsorption of BPA onto CD-based adsorbents has been extensively studied using various adsorption isotherm models. Among the reviewed studies, the most applied models include Langmuir, Freundlich, Sips, Redlich–Peterson, Halsey, Temkin, and Dubinin–Radushkevich (D-R) isotherms [2,3,4,8,9,10,11,12,48,65,68,69,70,71,72,73,74,75]. The Langmuir isotherm model, which assumes monolayer adsorption on a homogeneous surface, has been identified as the most suitable model in multiple studies [2,3,4,8,10,11,12,48,69,70,73]. In contrast, the Freundlich isotherm model, which describes adsorption on heterogeneous surfaces, has shown a better fit in certain cases, particularly when multilayer adsorption occurs [2,3,4,8,9,10,11,12,65,68,69,70,72]. The Sips isotherm model, a hybrid of Langmuir and Freundlich, has been tested on heterogeneous surfaces; however, in most studies, it exhibited a weaker fit than Langmuir [8,10,69,75]. The adsorption of BPA onto CD-based adsorbents has been best described by the Langmuir model in most studies. However, the Freundlich isotherm model provided better predictions for heterogeneous surfaces, particularly in systems exhibiting multilayer adsorption. While the Sips model successfully characterized heterogeneous surfaces in some cases, its fit was generally inferior to that of Langmuir. The Redlich–Peterson model, which integrates features of both Langmuir and Freundlich, has been tested in several studies, but it was rarely identified as the best-fitting model [3]. The Halsey and Temkin models have been examined in fewer studies and were not typically found to be the most appropriate models [72]. Overall, the Langmuir and Freundlich isotherms have been identified as the most suitable models for describing BPA adsorption onto CD-based adsorbents. However, the Sips and Redlich-Peterson models have shown applicability in certain heterogeneous adsorption conditions [72,75]. The Halsey and Temkin models exhibited limited applicability, being effective only under specific adsorption conditions [72].

**Figure 3:**
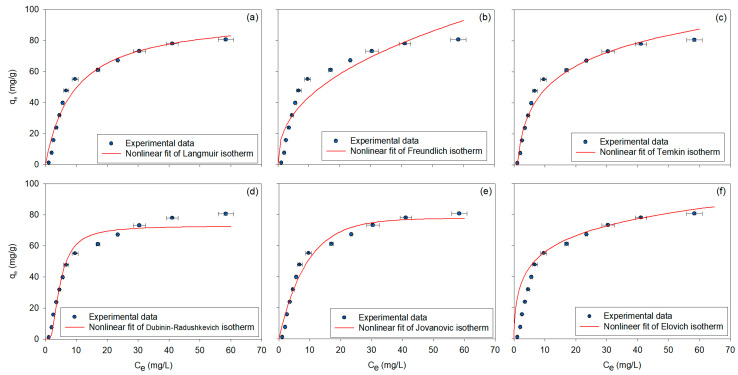
Nonlinear fitting of two-parameter monolayer adsorption isotherm models: (**a**) Langmuir isotherm, (**b**) Freundlich isotherm, (**c**) Temkin isotherm, (**d**) Dubinin-Radushkevich isotherm, (**e**) Jovanovic isotherm, (**f**) Elovich isotherm.

In this study, the adsorption behavior of BPA onto β-CDNSs was systematically evaluated using 12 different monolayer adsorption isotherm models [76]. These models were applied to analyze adsorption characteristics based on equilibrium adsorption data (C_e_, q_e_) obtained from the final equilibrium concentrations of BPA in solution with experiments conducted at _mβ-CDNS_ = 150 mg, N = 200 rpm, T = 25 °C. The nonlinear fitting results of the two-parameter adsorption isotherm models are presented in Figure 3a–f, with the corresponding calculated parameters listed in Table 1. The Langmuir isotherm [77] exhibited a high adsorption capacity of 95.8589 mg/g, suggesting that the adsorption followed a monolayer model on a homogeneous surface. The separation factor of 0.03–0.65 confirmed favorable adsorption, with no interaction between adsorbate molecules on the surface. The high model fit (R^2^ = 0.9629) suggested strong accuracy in predicting the adsorption process, confirming the Langmuir model’s suitability for describing adsorption under conditions of homogeneous surface properties and limited adsorption sites. These findings agree with studies where the Langmuir model provided the best fit for BPA adsorption on adsorbents like graphene oxide–β-CD and citric acid–crosslinked β-CD polymers [10,11]. The Freundlich isotherm [78], with an adsorption coefficient of 16.3742 (mg/g)(L/mg)^1−n^, indicated multilayer adsorption on a heterogeneous surface. The exponent value of 2.3596 confirmed favorable adsorption, while the reciprocal of the exponent suggested moderate surface heterogeneity. Although the fit (R^2^ = 0.8782) was slightly lower than that of the Langmuir model, it still effectively captured the adsorption behavior under non-homogeneous surface conditions, confirming the Freundlich model’s ability to describe multilayer adsorption on heterogeneous surfaces, as seen in studies like Gupta et al. (2017) [11] and Lukasik et al. (2023) [2]. The Temkin isotherm [79], with an adsorption heat-related parameter of 114.2393 J/mol and an equilibrium binding constant of 0.991 L/mg, suggested moderate interaction and a strong binding affinity between the adsorbate and adsorbent. The high model fit (R^2^ = 0.9736) emphasized that the adsorption energy varied across the surface, which is characteristic of systems where adsorption strength changes with concentration. These findings are consistent with Mamman et al. (2021), where the Temkin model was also found to be well-suited for modeling the adsorption of BPA onto hybridized cyclodextrin-based adsorbents, indicating that the adsorption energy is not uniform across the surface and depends on the surface characteristics [72]. The Dubinin–Radushkevich (D-R) isotherm [80], with a maximum adsorption capacity of 72.7681 mg/g, indicated that the adsorption process was predominantly physical, driven by van der Waals interactions. The mean free energy of 392.84 J/mol suggested a physical adsorption mechanism, consistent with the findings from Mamman et al. (2021), where the free energy values for BPA adsorption ranged between 0.2949 and 2.4011 kJ/mol, indicating a physisorption process. Values below 8 kJ/mol are typically associated with physical adsorption [72]. In our study, the D–R model showed a high fit to the equilibrium data (R^2^ = 0.9683), confirming the model’s effectiveness in describing the adsorption process. The Jovanovich isotherm [81], with a capacity of 77.4410 mg/g, suggested that the adsorption process exhibited a slower approach to saturation compared to the Langmuir model, indicating that the system reached gradual adsorption equilibrium. The moderate adsorption rate constant of 0.1105 L/mg reflected that adsorption reached equilibrium over time, as confirmed by the high model fit (R^2^ = 0.9680). These results align with findings in Łukasik et al. (2023) [2], where the Jovanovich model was found to effectively describe adsorption on adsorbents with slower kinetics. Finally, the Elovich isotherm [82], showing a maximum capacity of 20.1620 mg/g, supported the multilayer adsorption theory, particularly in systems with increasing adsorption site availability. The adsorption constant of 4.063 L/mg suggested a relatively strong interaction between the adsorbate and adsorbent, with a high fit (R^2^ = 0.9770) confirming the model’s applicability.

**Figure 4:**
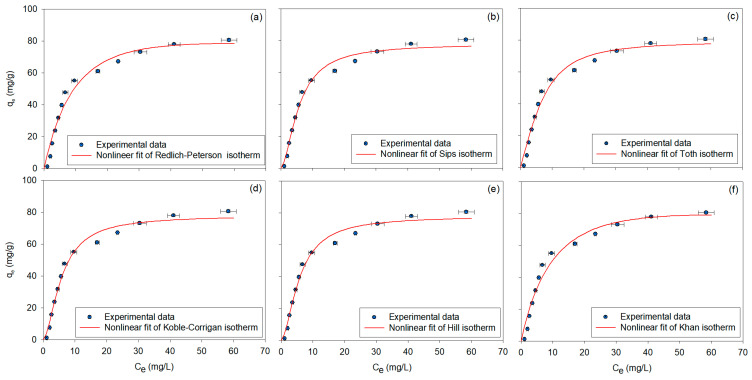
Nonlinear fitting of three-parameter monolayer adsorption isotherm models: (**a**) Redlich-Peterson isotherm, (**b**) Sips isotherm, (**c**) Toth isotherm, (**d**) Koble-Corrigan isotherm, (**e**) Hill isotherm, (**f**) Khan isotherm.

Similarly, the nonlinear fitting results of the three-parameter adsorption isotherm models are shown in Figure 4a–f, with the corresponding calculated parameters listed in Table 1. The Redlich–Peterson isotherm [83] provided a strong fit (R^2^ = 0.9629) and indicated a more complex interaction between the adsorbate and adsorbent. The exponent value (1.1813, slightly greater than 1) suggested a deviation from the Langmuir model, indicating that the adsorption system was more complex and did not follow ideal monolayer adsorption behavior. This is consistent with findings from Gong et al. (2020) [3], where the Redlich–Peterson isotherm was found to be the best fit for BPA adsorption onto Fe_3_O_4_@SiO_2_/chitosan/graphene oxide/β-cyclodextrin, suggesting multilayer adsorption on a heterogeneous surface with a strong agreement with experimental data (R^2^ ≈ 0.994). The Sips isotherm [84], with a maximum adsorption capacity of 77.8437 mg/g and a heterogeneity factor of 1.7050, confirmed significant surface heterogeneity and the system’s ability to transition between low-concentration Freundlich-like behavior and high-concentration Langmuir-like saturation. The high fit (R^2^ = 0.9861) indicated that the model effectively captured the adsorption process across various concentrations, demonstrating a strong agreement with experimental data. This finding is in line with Qu et al. (2023) [75], where the Sips model also showed high fitting accuracy (R^2^ = 0.9552) for heterogeneous systems, indicating a combination of Langmuir and Freundlich behavior. The Toth isotherm [85], with a capacity of 78.9506 mg/g, demonstrated strong adsorption potential and confirmed substantial surface heterogeneity, as indicated by the heterogeneity parameter (z = 1.9214). The high model fit (R^2^ = 0.9746) suggested that the Toth model effectively described the adsorption process across both low and high concentrations, outperforming the Sips model in representing adsorption behavior in this system. Although the Toth isotherm model has not been sufficiently examined in the studies, it can still be effectively applied to systems with varying adsorption energies to model complex adsorption behavior more accurately. The Koble–Corrigan isotherm [86], with a high model fit (R^2^ = 0.9861), described heterogeneous adsorption effectively. The heterogeneity factor (1.7090) indicated increased surface irregularity, confirming that the model was suitable for systems with varying adsorption energies. The Hill isotherm [87], with a maximum capacity of 77.8436 mg/g, exhibited positive cooperativity (n_H_ = 1.7050), meaning that binding at one site on the macromolecule increased the affinity at other sites. The dissociation constant of 19.9347 mg/L indicated that half of the available binding sites were occupied at this concentration, and the high fit (R^2^ = 0.9861) confirmed that the model effectively captured the cooperative adsorption mechanism. Finally, the Khan isotherm [88,89], with a maximum adsorption capacity of 153.2722 mg/g, demonstrated its flexibility in representing both Langmuir and Freundlich behaviors. The moderate adsorption strength constant (0.0603) and the exponent value of 1.2738 suggested multilayer adsorption at high concentrations, and the high model fit (R^2^ = 0.9670) further confirmed the model’s effectiveness in capturing complex adsorption systems.

Among the tested isotherm models, the Sips, Toth, and Koble–Corrigan models provided the most accurate predictions of the adsorption behavior, as evidenced by their high R^2^ values (above 0.97), confirming their robustness in modeling heterogeneous adsorption. The Khan model exhibited the highest adsorption capacity, indicating its strong applicability for high-concentration adsorption systems. The Langmuir and Jovanovich models remained effective for simpler, monolayer adsorption systems, while the Freundlich and Elovich models were more suitable for multilayer adsorption behavior. The Hill and Redlich–Peterson models captured cooperative binding mechanisms and adsorption behaviors, particularly for systems with varying surface energies. Overall, the evaluation of all twelve isotherm models highlights the complex nature of BPA adsorption onto β-CDNS polymers, demonstrating that no single model can universally describe the entire adsorption process. Instead, the selection of an appropriate isotherm depends on the specific adsorption conditions, surface properties, and concentration ranges, emphasizing the need for model comparison in adsorption studies.

#### 3.2.2. Adsorption Kinetics and Adsorption Mechanism

**Figure 5:**
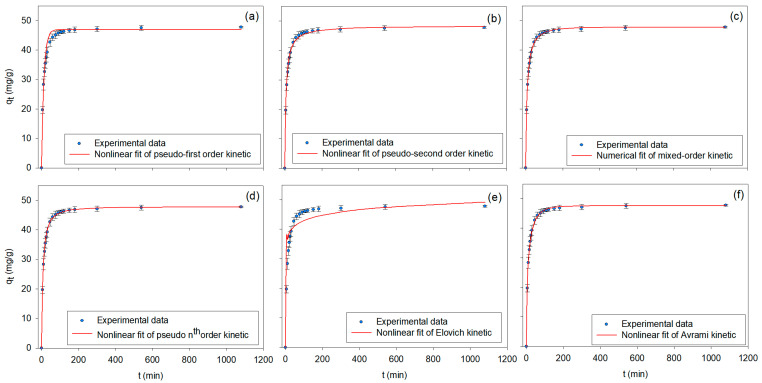
Nonlinear and numerical fitting of adsorption kinetic models: (**a**) pseudo-first order kinetic, (**b**) pseudo-second order kinetic, (**c**) mixed-order kinetic, (**d**) pseudo-nth order kinetic, (**e**) Elovich kinetic, (**f**) Avrami kinetic.

The adsorption kinetics of BPA onto β-CDNS were analyzed using several models: pseudo–first order (PFO), pseudo–second order (PSO), mixed order (MO), pseudo–nth order (PNO), Elovich, and Avrami models [90]. The experiments were conducted under the following conditions: C_0_ = 150 mg/L, m_β-CDNS_ = 0.15 g, N = 200 rpm, and T = 25 °C. The kinetic fitting results, including the nonlinear forms of the models, are presented in Table 2, with experimental data and the corresponding best-fit models illustrated in Figure 5a–f. The results show that the BPA adsorption process is complex, involving heterogeneous surface interactions and multi-step kinetics. Notably, the MO and PNO models indicated a multi-step interaction involving surface sites with varying energy levels, emphasizing the heterogeneous nature of the adsorption surface. These findings are consistent with the findings reported by Bucur et al. (2022), who observed that adsorption onto β-CD–modified materials involves multiple steps, with different active sites contributing to the adsorption process [12]. Additionally, the excellent fit of the PSO model supports the dominance of chemical interactions, aligning with findings from Gupta et al. (2017), who found that chemical adsorption is the predominant mechanism in BPA adsorption onto β-CD–based adsorbents [11].

The MO and PNO models exhibited high R^2^ values (MO: R^2^ = 0.9996, PNO: R^2^ = 0.9991), suggesting that the adsorption follows a combination of PFO and PSO kinetics. This finding agrees with the observations of Gupta et al. (2017) and Gong et al. (2020), where adsorption was determined to involve multi-step kinetics, indicating that the surface is heterogeneous with different types of adsorption sites [3,11] The PSO model demonstrated the best fit (R^2^ = 0.9977), confirming that chemical adsorption is the dominant process. Mamman (2021) also found that the PSO model provides the best fit for chemical adsorption, suggesting that valence forces or electron sharing lead to the strong bonding of BPA to the adsorbent surface [72]. The PFO model provided a weaker fit compared to the PSO, MO, and PNO models, with a lower R^2^ value (R^2^ = 0.9739). This suggests that physisorption alone cannot explain the adsorption behavior of BPA. This finding aligns with Łukasik et al. (2023), who found that the PFO model often underperforms in explaining adsorption processes, especially when chemical interactions dominate [2]. The Elovich model showed the poorest correlation (R^2^ = 0.8426), suggesting that surface heterogeneity and diffusion limitations are not the main factors controlling the adsorption in this system, which is consistent with the findings of Bucur et al. (2022) [12]. The Avrami model (R^2^ = 0.9997) indicated a complex adsorption pathway, likely involving nucleation and growth mechanisms, which is supported by findings from Mamman (2021), where Avrami was found to effectively describe multi-step kinetic mechanisms [72].

**Figure 6:**
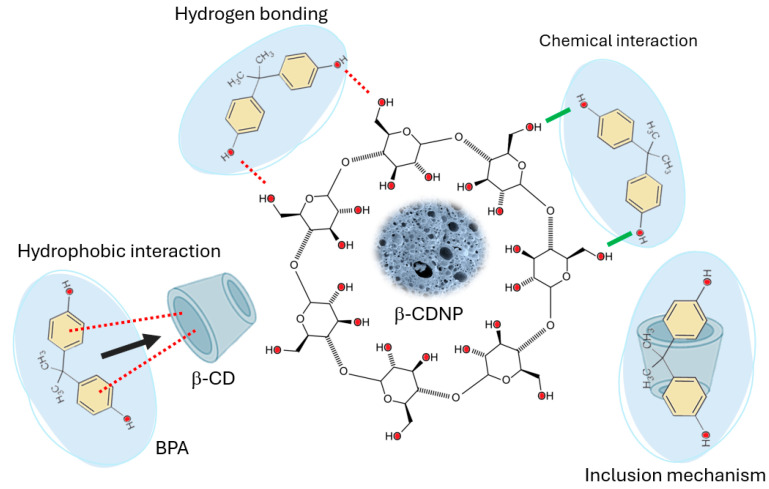
BPA adsorption mechanisms onto β-CDNS.

The BPA adsorption mechanism can be explained by a combination of three primary interactions, as illustrated in Figure 6.

Initially, BPA molecules are encapsulated in the hydrophobic cavities of β-CD, facilitating the early stages of adsorption through host–guest interactions. Studies by Bucur et al. (2022) and Gupta et al. (2017) have highlighted the importance of these interactions in enhancing BPA’s affinity for β-CD–based adsorbents [11,12]. As shown in Figure 6, due to its hydrophobic nature, BPA also interacts strongly with the hydrophobic regions of the adsorbent surface, as emphasized by Gupta et al. (2017) and Mamman et al. (2021), further driving the overall adsorption process [11,72]. In the final stage, hydrogen bonding between BPA’s hydroxyl groups and the β-CD surface contributes to the chemical nature of adsorption, leading to chemisorption. This involves the formation of strong chemical bonds, likely through electron sharing or valence forces, as suggested by Huang et al. (2018) and Gong et al. (2020) [3,10]. The dominance of chemisorption is confirmed by the PSO model, which indicates that the adsorption is primarily driven by chemical interactions, a conclusion supported by Bucur et al. (2022) and Łukasik et al. (2023) [2,12]. The adsorption process is multi-step and involves heterogeneous surface sites with varying energies, as indicated by the excellent fit of the MO and PNO models, underscoring the complexity of the adsorption kinetics.

Overall, the kinetic modeling results demonstrate that the BPA adsorption process is complex, involving multi-step interactions and heterogeneous surface interactions, with chemical interactions being dominant. These findings are further supported by the thermodynamic data presented in “Section 3.2.3 Adsorption Thermodynamics”. The thermodynamic analysis reveals that the adsorption process is exothermic and spontaneous; however, the adsorption efficiency decreases with increasing temperature. The kinetic and thermodynamic analyses together highlight that the adsorption mechanism is a complex process driven by a combination of both physical and chemical interactions.

#### 3.2.3. Adsorption Thermodynamics

The thermodynamic feasibility of BPA adsorption onto β-CDNSs is summarized in Table 3. The experiments were conducted under the following conditions: C_0_ = 150 mg/L, m_β-CDNS_ = 0.15 g, and N = 200 rpm. These parameters were derived from the van’t Hoff and Gibbs equations. ΔH^o^ and ΔS^o^ were obtained through the linear regression of the van’t Hoff equation, as presented in Equation (6), with an R^2^ value of 0.9805, while ΔG^o^ was calculated using Equation (5).

The negative ΔH^o^ value confirmed the exothermic nature of the adsorption process, indicating that BPA molecules interacted with the β-CDNSs primarily via physisorption, characterized by van der Waals forces and hydrogen bonding. The enthalpy change (ΔH° = −11.73 kJ/mol) falls within the characteristic range of physisorption (4–40 kJ/mol), reinforcing that the adsorption mechanism is dominated by non-covalent interactions. As adsorption releases energy due to its exothermic nature, it becomes less favorable at higher temperatures, a trend commonly observed in physisorption-driven systems. The negative ΔS^o^ indicates a decrease in system randomness, implying that BPA molecules become more ordered upon interaction with the nanosponge structure, likely due to host–guest complexation within the β-CD cavities. Given that van der Waals interactions typically exhibit ΔH° values within 4–10 kJ/mol and hydrogen bonding within 10–40 kJ/mol, the obtained enthalpy change suggests that hydrogen bonding is the dominant interaction type in the adsorption process. The negative ΔG° values at all investigated temperatures confirm that adsorption is thermodynamically favorable and spontaneous, with a decreasing trend at higher temperatures, reflecting a decline in adsorption efficiency due to the weakening of intermolecular forces. This behavior aligns with the observed ΔH° and ΔS° values, supporting the conclusion that the adsorption mechanism is governed by physical interactions, although chemisorption can contribute at higher temperatures.

Comparison with previous studies shows that thermodynamic investigations on BPA adsorption onto CD-based materials consistently demonstrate their spontaneous nature, as evidenced by negative ΔG° values [8,72,74]. Most studies suggest an exothermic process, where adsorption becomes less favorable at higher temperatures, with enthalpy values ranging from −40.92 kJ/mol [91] to −18.91 kJ/mol [72]. However, some studies observed endothermic behavior where adsorption increased with temperature [74]. Entropy change (ΔS°) varied across studies, with some reporting negative values. For instance, Mphahlele et al. (2015) [91] reported an entropy change of −125.00 J/molK, while Mamman et al. (2021) [72] observed −51.72 J/mol K, indicating a more ordered adsorption system. In contrast, other studies [8,74] found positive ΔS° values, suggesting increased randomness at the solid–liquid interface. These findings confirm that BPA adsorption onto CDs is feasible, predominantly exothermic, and influenced by both physisorption and chemisorption, depending on temperature conditions.

### 3.3. Supercritical CO_2_-Based Green Regeneration

**Figure 7:**
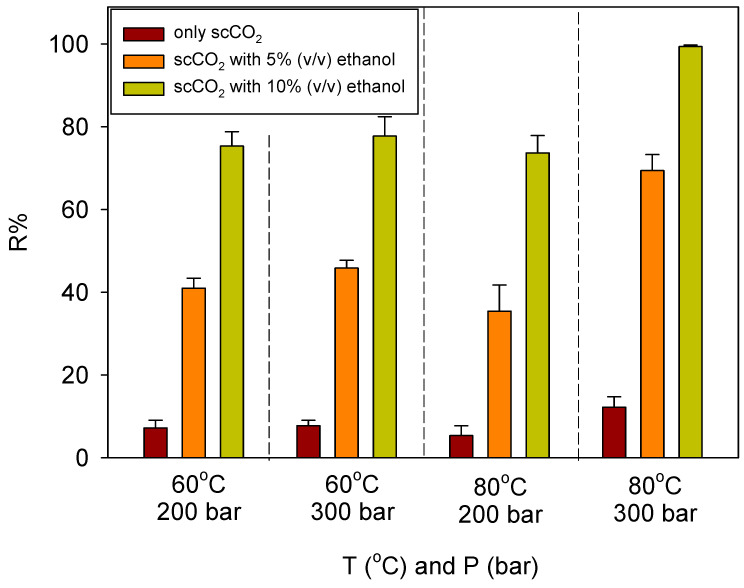
Effect of temperature, pressure, and ethanol concentration on regeneration efficiency (R%) in scCO_2_-based green regeneration after 3 h.

The effect of scCO_2_-based green regeneration conditions on regeneration efficiency (R%) is shown in Figure 7. Regeneration performed using only scCO_2_ resulted in a maximum efficiency of 12.22 ± 2.49%. In contrast, in the experiments with 5% (*v*/*v*) ethanol added to scCO_2_, R% values reached a minimum of 35.42 ± 6.34%, indicating a significant improvement. Additionally, BPA exhibits extremely low solubility in scCO_2_ (~10^−^⁵ mole fraction), whereas its solubility significantly increases in ethanol (~10^−2^ mole fraction) [92,93]. At constant temperature, the R% values increased due to the increase in pressure. The underlying reason for this phenomenon is that, as pressure increases at constant temperature, the density of the supercritical fluid also increases. This is because the dissolving power of the supercritical fluid is directly proportional to its density. In this study, the dissolving power of the supercritical fluid in the recovery of BPA was significantly enhanced by the addition of 5% (*v*/*v*) and 10% (*v*/*v*) ethanol, acting as a polar co-solvent, into scCO_2_. Under conditions other than 80 °C and 300 bar, the regeneration efficiency values ranged from 35.42 ± 6.34% to 45.85 ± 1.88% with 5% (*v*/*v*) ethanol. However, with 10% (*v*/*v*) ethanol, the values increased by nearly 1.7 to 2.0 times, ranging from 73.70 ± 4.19% to 77.75 ± 4.69%. The most effective regeneration values were achieved at 80 °C and 300 bar, reaching 69.42 ± 3.87% with 5% (*v*/*v*) ethanol and 99.40 ± 0.35% with 10% (*v*/*v*) ethanol. The result shows that the regeneration efficiency in the scCO_2_ system with 10% ethanol at 300 bar increases from 77.75 ± 4.69% at 60 °C to 99.40 ± 0.35% at 80 °C. This increase was due to the enhanced solubility of scCO_2_ with rising temperature. The addition of ethanol further boosted the solubility of scCO_2_, improving the process. This behavior can be attributed to a cross-over effect [56], where, typically, increasing temperature leads to a decrease in solubility due to the reduced density of scCO_2_. However, the presence of ethanol as a co-solvent counteracts this effect by increasing the solubility of BPA, allowing for higher desorption efficiency. As a result, the combination of high temperature, high pressure, and ethanol significantly enhances R%, illustrating the importance of optimizing these parameters to achieve maximum BPA recovery.

Building upon these findings, it is evident that the addition of ethanol significantly enhances the regeneration efficiency of BPA in scCO_2_-based systems. Ethanol, a polar co-solvent, increases the solubility of BPA in scCO_2_, which otherwise has limited ability to dissolve this moderately polar compound. The enhanced solubility and improved desorption are attributed to ethanol’s role in modifying intermolecular interactions, reducing interfacial tension, and increasing the diffusion coefficient of BPA, leading to a more efficient mass transfer process and higher regeneration efficiencies, particularly with increased ethanol concentrations. Both temperature and pressure further influence the system’s effectiveness. Temperature impacts the viscosity of scCO_2_ and the diffusivity of BPA within the sorbent matrix, while pressure increases the density of the supercritical fluid, improving its solvating power and promoting better interaction with BPA molecules. However, beyond a certain pressure, further increases may have diminishing returns. The combination of 10% ethanol with scCO_2_ at 80 °C and 300 bar yielded the highest regeneration efficiency, reaching nearly 99.4%. This demonstrates that optimizing the balance of temperature, pressure, and ethanol concentration significantly enhances BPA recovery, making this process more efficient and environmentally sustainable compared to conventional methods. In conventional BPA regeneration processes using CD-based polymers, acetone and ethanol-HCl mixtures are typically employed, achieving BPA recovery rates of 90% to 94%. After regeneration, BPA is desorbed from the adsorbent and transferred into the ethanol phase, resulting in a BPA-containing ethanol solution. A promising method for separating BPA from ethanol is through controlled depressurization or rapid expansion of supercritical solutions (RESSs), where a sudden pressure drop reduces BPA solubility in ethanol, causing precipitation [94]. This solvent-free separation technique minimizes chemical processing and waste generation. Additionally, crystallization can be used for selective recovery, where BPA precipitates upon cooling, leaving ethanol for reuse. Supercritical CO_2_, known for its recyclability, is an environmentally sustainable solvent. Industrial systems can achieve 90–98% recovery efficiency by collecting, recompressing, and reusing CO_2_, significantly reducing the need for fresh CO_2_ and aligning with green chemistry principles. This study employed a 3 h regeneration period, ensuring sufficient mass transfer for complete desorption, with previous studies [56] confirming that phenolic compounds, including BPA, reach optimal desorption efficiency within this time frame. Further investigations are needed to determine the ideal regeneration duration, as longer periods may increase energy consumption without significant additional benefits. Fine-tuning ethanol concentration and regeneration time can also improve cost-effectiveness and scalability, as shown by the significant impact of ethanol concentration on desorption efficiency. The findings suggest that a careful balance between ethanol concentration, temperature, pressure, and regeneration time is essential for optimizing the process. Further studies should explore the scalability of this method for industrial applications, ensuring that it remains energy-efficient and environmentally friendly while maintaining high recovery efficiency.

**Figure 8:**
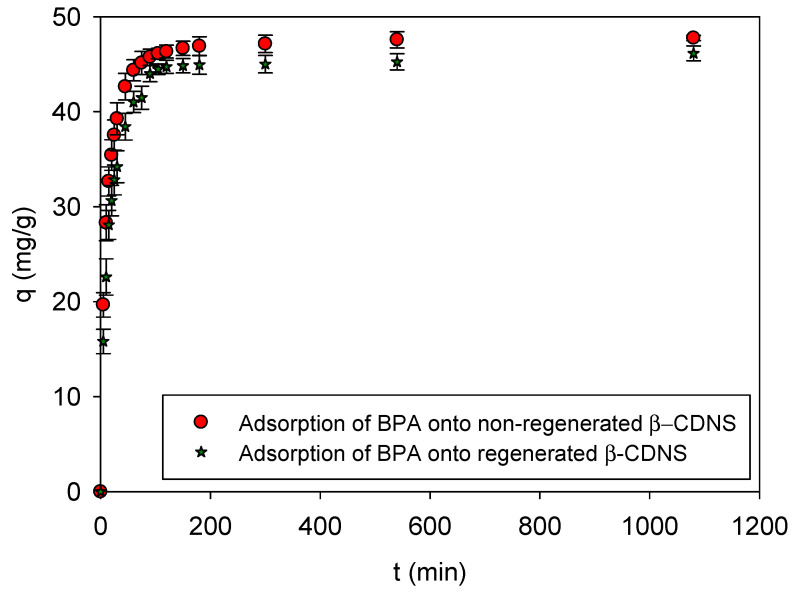
BPA adsorption kinetics onto non-regenerated and regenerated β-CDNS.

The adsorption kinetics of BPA onto both non-regenerated and regenerated β-CDNSs are presented in Figure 8. The results indicate that BPA adsorption occurred rapidly within the first 30 min due to the availability of abundant active sites on the adsorbent surface. Over 90% of total adsorption was completed within the first 60 min, after which the rate gradually slowed as equilibrium was approached, consistent with a PSO kinetic model. At equilibrium (t = 120 min), the non-regenerated β-CDNS achieved an adsorption capacity of 46.33 ± 0.68 mg/g, while the regenerated β-CDNS exhibited a slightly lower capacity of 44.73 ± 0.68 mg/g. Despite this minor difference (<5%), the regenerated material maintained a high adsorption performance even at extended adsorption times (t = 1080 min), reaching 46.13 ± 0.78 mg/g. These findings demonstrate that scCO_2_-based green regeneration effectively preserved the structural integrity and adsorption efficiency of β-CDNS, making it a viable and sustainable method for reuse. The slight reduction in adsorption capacity does not significantly impact its practical application, reinforcing its suitability for long-term environmental remediation and wastewater treatment efforts.

### 3.4. Structural and Functional Characterization of β-CDNSs

#### 3.4.1. FTIR Characterization and Molecular Interactions of β-CDNSs

**Figure 9:**
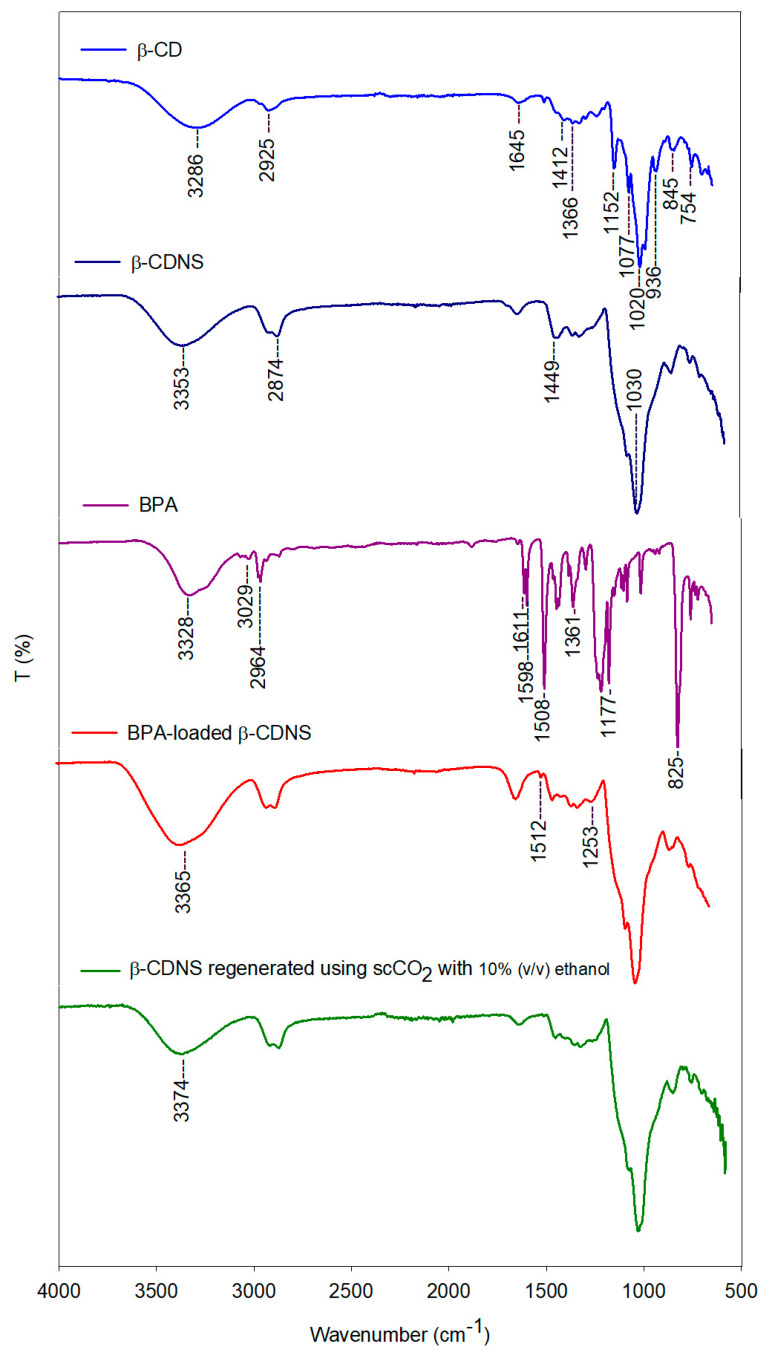
FTIR spectra of β-CD, β-CDNS, BPA, BPA-loaded β-CDNS under optimized adsorption conditions, and β-CDNS regenerated by scCO_2_-based green regeneration process (300 bar, 80 °C, scCO_2_ with 10% (*v*/*v*) ethanol, 3 h).

The FTIR spectral analysis (Figure 9) provided comprehensive insights into the structural properties and molecular interactions of β-CD, β-CDNS, BPA, BPA-loaded β-CDNS under optimized adsorption conditions, and β-CDNS regenerated by the scCO_2_-based green regeneration process (300 bar, 80 °C, scCO_2_ with 10% (*v*/*v*) ethanol, 3 h).

The FTIR spectrum of β-CD exhibited characteristic vibration bands, including O-H stretching vibration at 3286 cm^−1^, C-H stretching vibration at 2925 cm^−1^, H-OH bending vibration at 1645 cm^−1^, C-H deformation vibrations at 1412 cm^−1^ and 1366 cm^−1^, C-C stretching vibration at 1152 cm^−1^, C-O stretching vibration at 1077 cm^−1^, and C-O-C stretching vibration at 1020 cm^−1^. Additionally, glucopyranose-associated stretching vibrations were observed at 936 cm^−1^, 845 cm^−1^, and 754 cm^−1^, confirming the expected spectral characteristics of β-CD [95]. In the FTIR spectrum of β-CDNS, the O-H stretching vibration at 3353 cm^−1^, the C-H stretching vibration at 2874 cm^−1^, the C-H bending vibration at 1449 cm^−1^, and the C-O-C stretching vibration at 1030 cm^−1^, attributed to the normal alkane structure, were observed. The similarities between the spectra of the original β-CD and β-CDNS indicate that the basic structural units were preserved in β-CDNS. However, some bands exhibited shifts or broadening, confirming that a crosslinking reaction occurs between β-CD and EPI, leading to the formation of new chemical bonds [96]. Specifically, the increased intensity of the O-H stretching vibration at 3353 cm^−1^ suggests the generation of new O-H groups during the crosslinking process. Additionally, the intensities of the C-H stretching vibrations at 2874 cm^−1^ and 1449 cm^−1^, as well as the C-O-C stretching vibration at 1030 cm^−1^, also increased, further supporting the successful formation of crosslinked β-CDNS [58]. The FTIR spectrum of BPA displayed a broad asymmetric O-H stretching vibration at 3328 cm^−1^ due to self-H–bonded O-H groups, an aromatic C-H stretching vibration at 3029 cm^−1^, and a characteristic CH_3_ asymmetric stretching vibration at 2964 cm^−1^. Additionally, the para-substituted aromatic system produced well-defined vibration bands at 1611, 1598, 1508, 1177, and 825 cm^−1^, while the isopropyl structure was associated with a weak vibration band at 1361 cm^−1^ [97]. Furthermore, the O-H stretching vibration band between 3700 cm^−1^ and 3000 cm^−1^ in β-CDNS broadened after BPA adsorption, and the stretching frequency of the O-H group exhibited a slight shift from 3353 cm^−1^ to 3365 cm^−1^. This shift can be attributed to hydrogen bonding interactions between hydroxyl groups present in both BPA and β-CDNS. Sirach and Dave (2023) reported that the –OH group plays a crucial role in the formation of hydrogen bonds between the adsorbent and BPA, thereby facilitating the adsorption of organic pollutants [9]. After BPA adsorption, no significant changes were observed in the FTIR spectrum of β-CDNS; however, new peaks appeared at 1512 cm^−1^ and 1253 cm^−1^, which corresponded to those in the FTIR spectrum of BPA. These findings confirm the successful incorporation of BPA molecules into the β-CDNS structure. Additionally, the O-H stretching vibration band between 3700 cm^−1^ and 3000 cm^−1^ in β-CDNS exhibited broadening upon BPA adsorption, further supporting the role of hydrogen bonding in the interaction.

Sirach and Dave (2023) highlighted that hydroxyl groups play a key role in hydrogen bond formation between the adsorbent and BPA, thereby enhancing the adsorption of organic pollutants [9]. In the FTIR spectrum of regenerated β-CDNS after BPA adsorption, the characteristic vibration bands of BPA at 1512 cm^−1^ and 1253 cm^−1^ disappeared, indicating the near-complete removal of BPA from the β-CDNS structure and confirming the overall success of the regeneration process. Additionally, the broadening observed at 3365 cm^−1^ in the FTIR spectrum of BPA-adsorbed β-CDNS was significantly reduced, resulting in a spectral profile similar to that of the original β-CDNS. This confirms the efficiency of the regeneration process, as the adsorbed BPA was successfully eliminated, restoring the structure to its initial state.

#### 3.4.2. Thermal Characterization of β-Cyclodextrin Nanosponges via DSC

**Figure 10:**
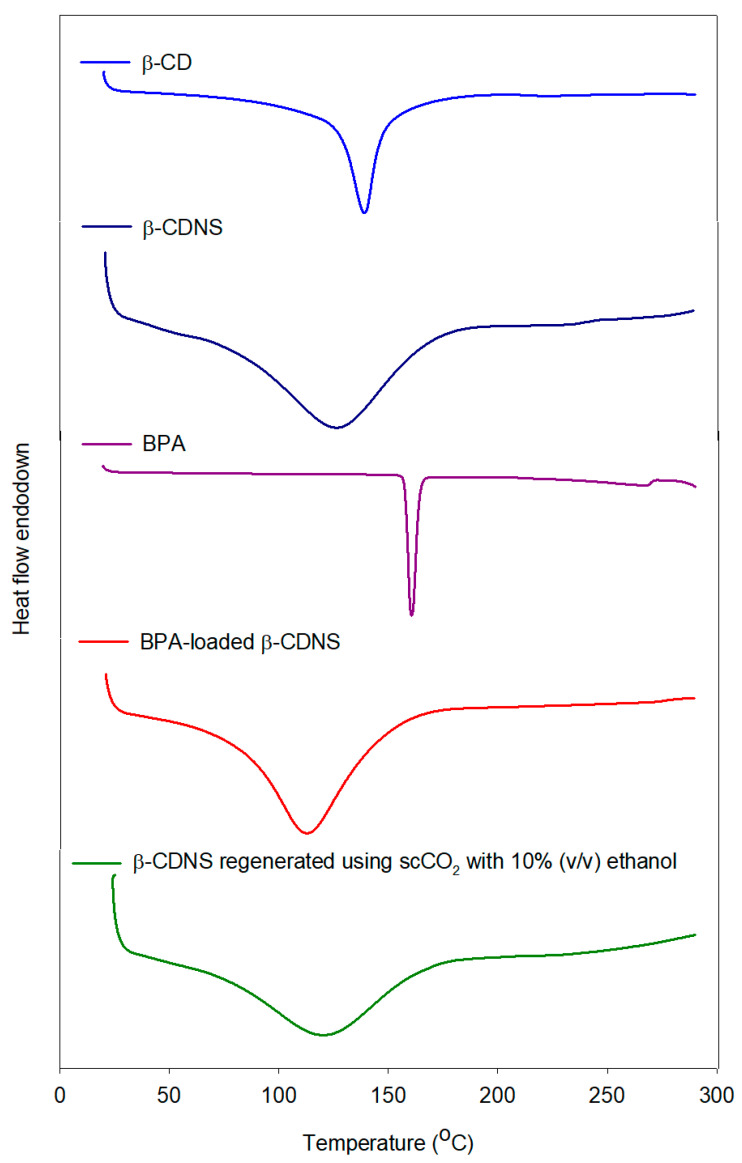
DSC thermograms of β-CD, β-CDNS, BPA, BPA-loaded β-CDNS under optimized adsorption conditions, and regenerated β-CDNS by scCO_2_-based green regeneration process (300 bar, 80 °C, scCO_2_ with 10% (*v*/*v*) ethanol, 3 h).

The DSC analysis (Figure 10) of β-CD, β-CDNS, BPA, BPA-loaded β-CDNS, and regenerated β-CDNS up to 290 °C provided crucial insights into their thermal behavior and structural stability.

The first major endothermic event, observed between 100 °C and 140 °C in β-CD, corresponds to the loss of bound water molecules, whereas in β-CDNS, this transition shifts to 130–160 °C and broadens, suggesting reduced free water content due to crosslinking, thereby enhancing thermal stability. BPA exhibited a distinct melting peak from 155 °C to 170 °C, which remained present in the BPA-loaded β-CDNS complex, indicating successful adsorption and potential host–guest interactions. The adsorption of BPA onto β-CDNS leads to broadening and slight shifts in the BPA melting peak, likely resulting from hydrogen bonding and hydrophobic interactions. The DSC thermogram of regenerated β-CDNS closely resembled that of pristine β-CDNS, with a significant reduction or complete disappearance of the BPA-associated endothermic event, confirming the effective removal of BPA through supercritical regeneration. However, the dehydration peak at 130–160 °C persisted, indicating that the structural integrity of β-CDNS was largely preserved post-regeneration. These findings demonstrate that, while BPA adsorption altered the thermal properties of β-CDNS, the regeneration process effectively restored its original characteristics, supporting its potential for repeated adsorption–regeneration cycles.

#### 3.4.3. Morphological Characterization of β-Cyclodextrin Nanosponges via SEM

**Figure 11:**
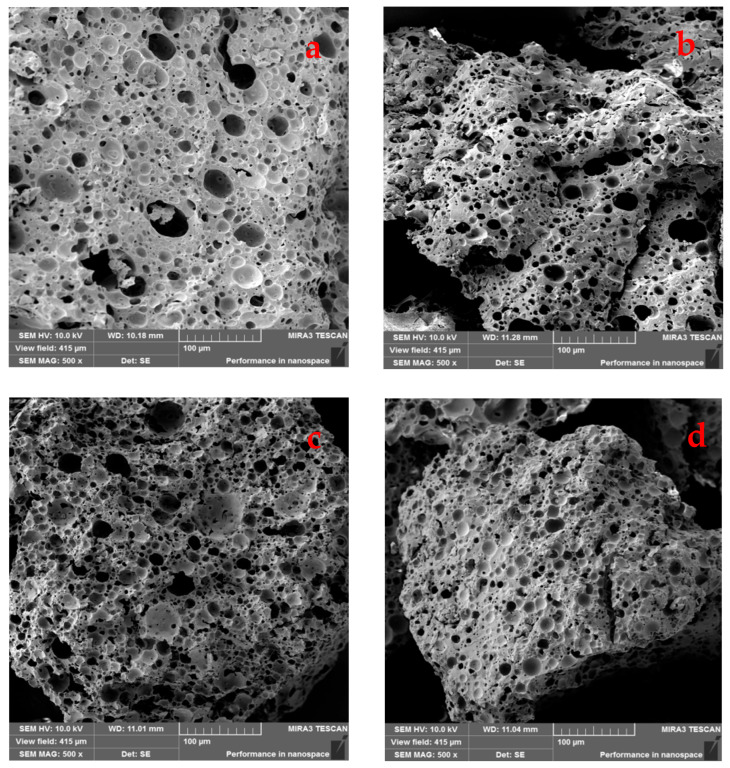
SEM images of β-CDNS at different stages: (**a**) β-CDNS before grinding, (**b**) β-CDNS after grinding, (**c**) BPA-loaded β-CDNS under optimized adsorption conditions, and (**d**) β-CDNS regenerated by scCO_2_-based green regeneration process (300 bar, 80 °C, scCO_2_ with 10% (*v*/*v*) ethanol, 3 h).

The SEM analysis (Figure 11) revealed significant morphological transformations in β-CDNS during the adsorption and regeneration processes utilizing supercritical green solvents. Initially, β-CDNS exhibited an irregular morphology with high porosity (45.2%), which decreased after grinding (41.1%), resulting in a more compact and uniform structure. BPA adsorption further reduced porosity (40.7%), indicating partial pore occlusion. Supercritical green solvent regeneration effectively restored porosity to the grinding stage level (41.1%), demonstrating its efficiency in maintaining structural integrity. Surface roughness increased after grinding (~40%) and peaked following BPA adsorption (~55%), likely due to molecular deposition. After supercritical green solvent regeneration, roughness values returned to pre-adsorption conditions, suggesting the successful removal of adsorbed molecules while preserving surface characteristics. Fractal dimension analysis indicated greater surface complexity after grinding, whereas BPA loading slightly reduced complexity, leading to a more uniform surface. Pore shape analysis confirmed that grinding improved shape regularity, while adsorption and supercritical green solvent-based regeneration had distinct effects on pore distribution. Overall, these findings highlight the impact of processing conditions on adsorption efficiency and material reusability. Supercritical green solvent regeneration proved more effective at preserving structural heterogeneity and adsorption potential, supporting its suitability for maintaining the functionality of β-CDNS.

#### 3.4.4. Particle Size Distribution and Temporal Stability of β-CDNSs

**Figure 12:**
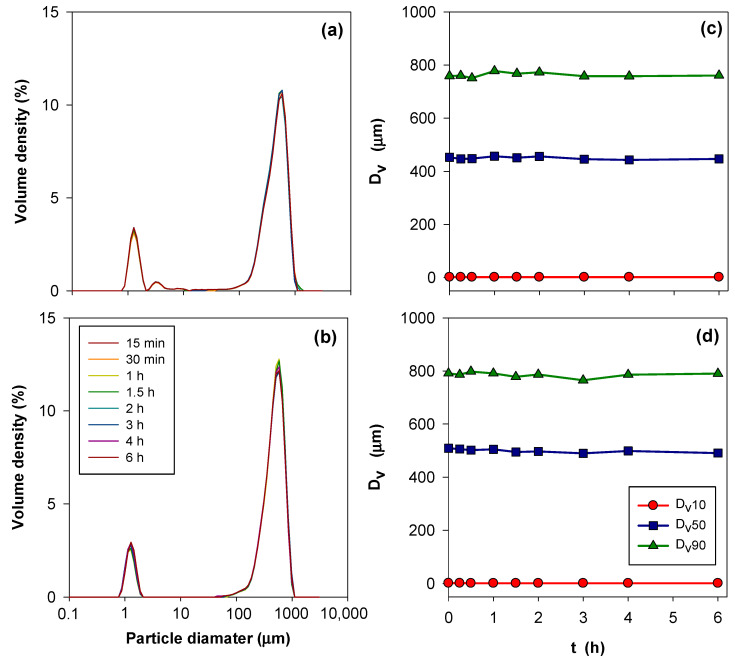
Particle size distributions of original β-CDNSs (**a**) before and (**b**) after regeneration at different retention times, with (**c**,**d**) Dv values indicating slight increases post-regeneration.

As shown in Figure 12, the particle size distributions and Dv values of the β-CDNSs were evaluated to assess their stability before and after the adsorption and regeneration processes. In Figure 12a, the particle size distributions of untreated β-CDNSs at different retention times (15 min to 6 h) remained consistent, indicating no signs of agglomeration, swelling, or dissolution. These findings align with our previous study [64], which demonstrated that the particle size distribution remained stable over time. The Dv values for untreated β-CDNSs were Dv10 = 1.59 µm ± 0.01, Dv50 = 449.78 µm ± 4.49, and Dv90 = 762.89 µm ± 7.98, confirming their structural stability during ultrasonication and mechanical stirring. Additionally, sieving analysis indicated that β-CDNS particles within the 150–500 µm range exhibited medium to low sphericity, confirming their irregular morphology. This irregularity is likely due to mechanical forces applied during grinding with an agate mortar and pestle.

In Figure 12b, the particle size distributions of β-CDNSs after adsorption and supercritical fluid regeneration exhibited minimal variation, demonstrating morphological stability. As depicted in Figure 12c,d, the Dv values (Dv10, Dv50, and Dv90) increased slightly post-regeneration to Dv10 = 1.77 µm ± 0.11, Dv50 = 499.33 µm ± 6.31, and Dv90 = 785.78 µm ± 8.92. These results suggest that β-CDNSs retained their structural integrity, making them suitable for repeated adsorption–regeneration cycles, while indicating minor surface alterations or slight agglomeration. The 3% increase in Dv90 was negligible, whereas the approximately 11% increase in Dv10 and Dv50 may be relevant depending on measurement accuracy and application requirements.

## 4. Conclusions

This study systematically investigated the adsorption behavior of BPA onto β-CDNSs and explored an innovative scCO_2_-based green regeneration process as a sustainable adsorbent recovery method. The adsorption process demonstrated exceptional efficiency, achieving a removal rate of 95.51 ± 0.82% under optimal conditions. Through nonlinear regression and numerical optimization techniques, comprehensive isotherm, kinetic, and thermodynamic analyses provided deep insights into the adsorption mechanism, revealing that the process was primarily governed by a chemisorption-dominated pseudo–second order model, with strong host–guest interactions facilitated by hydrogen bonding.

The scCO_2_-based green regeneration strategy exhibited remarkable efficiency, successfully restoring over 99% of the adsorption capacity under optimized conditions (300 bar, 60 °C, 10% (*v*/*v*) ethanol). Structural and thermal characterization confirmed that the β-CDNSs maintained their integrity after the adsorption–regeneration cycle, underscoring their potential for repeated use in wastewater treatment. The findings not only highlight the superior adsorption performance of β-CDNSs but also emphasize the sustainability of the regeneration process, which eliminates the need for hazardous solvents, reduces secondary waste production, and enhances the longevity of the adsorbent.

Given the increasing concerns over endocrine-disrupting chemicals in aquatic environments, this study provides a compelling foundation for the implementation of β-CDNS–based adsorption systems in real-world wastewater treatment applications. The integration of computational modeling, experimental optimization, and green regeneration methods demonstrates a viable pathway for developing next-generation adsorbents that align with global sustainability goals. Future research should focus on scaling up the process, evaluating long-term adsorption–regeneration performance, and exploring hybrid treatment approaches that combine adsorption with advanced oxidation or membrane-based technologies for enhanced pollutant removal.

Ultimately, this research contributes to the advancement of eco-friendly and high-efficiency adsorbents, offering a promising solution for mitigating BPA contamination and fostering environmental sustainability in industrial wastewater management.

## Figures and Tables

**Table 1:** Adsorption isotherm fitting parameters for BPA adsorption onto β-CDNS.

**Isotherm**	Equation	Model Parameters			$R^{2}$	Ref.
Two-parameter monolayer adsorption isotherm models						
Langmuir	$q_{e}=\frac{q_{mL}K_{L}C_{e}}{1+K_{L}C_{e}}$	$q_{mL}$	$K_{L}$			[78]
		95.8589	0.1068		0.9629	
Freundlich	$q_{e}=K_{F}C_{e}^{1/n_{F}}$	$K_{F}$	$n_{F}$			[79]
		16.3742	2.3596		0.8782	
Temkin	$q_{e}=\frac{RT}{B_{T}}ln(K_{T}C_{e})$	$B_{T}$	$K_{T}$			[80]
		114.2393	0.9391		0.9736	
Dubinin–Radushkevich (D–R)	$q_{e}=q_{DR}e^{-K_{DR}\varepsilon^{2}}$	$q_{DR}$	$K_{DR}$			[81]
		72.7684	3.24 × 10^−6^		0.9683	
Jovanovic	$q_{e}=q_{mJ}\left(1-e^{-K_{J}C_{e}}\right)$	$q_{mJ}$	$K_{J}$			[82]
		77.4110	0.1105		0.9680	
Elovich	$C_{e}=\frac{q_{e}}{q_{mE}K_{E}e^{\left(-\frac{q_{e}}{q_{mE}}\right)}}$	$q_{mE}$	$K_{E}$			[83]
		20.1620	4.0600		0.9770	
Three-parameter monolayer adsorption isotherm models						
Redlich–Peterson	$q_{e}=\frac{K_{RP}C_{e}}{1+a_{RP}C_{e}^{g}}$	$K_{RP}$	$a_{RP}$	g		[84]
		8.5450	0.0440	1.1813	0.9629	
Sips	$q_{e}=\frac{q_{mS}a_{S}C_{e}^{B_{S}}}{1+a_{S}C_{e}^{B_{S}}}$	$q_{mS}$	$a_{S}$	$B_{S}$		[85]
		77.8437	0.0502	1.7050	0.9861	
Toth	$q_{e}=\frac{q_{mT}C_{e}}{{\left(a_{T}+C_{e}^{z}\right)}^{1/z}}$	$q_{mT}$	$a_{T}$	z		[86]
		78.9506	94.1044	1.9214	0.9746	
Koble–Corrigan	$q_{e}=\frac{AC_{e}^{n_{K}}}{1+BC_{e}^{n_{K}}}$	A	B	$n_{K}$		[87]
		3.9049	0.0502	1.7050	0.9861	
Hill	$q_{e}=\frac{q_{SH}C_{e}^{n_{H}}}{K_{D}+C_{e}^{n_{H}}}$	$q_{SH}$	$K_{D}$	$n_{H}$		[88]
		77.8436	19.9347	1.7050	0.9861	
Khan	$q_{e}=\frac{a_{SK}b_{K}C_{e}}{{\left(1+b_{K}C_{e}\right)}^{a_{K}}}$	$a_{SK}$	$b_{K}$	$a_{K}$		[89,90]
		153.2722	0.0603	1.2738	0.9670	

[a_K_: Khan model exponent, a_RP_: Redlich–Peterson isotherm constant, a_S_: Sips equilibrium constant, a_T_: Toth equilibrium constant, A: Koble–Corrigan constant, b_K_: Khan constant, B: Koble–Corrigan constant, B_S_: Sips model exponent, B_T_: Temkin isotherm constant, C_e_: equilibrium concentration of adsorbate, g: Redlich–Peterson isotherm exponent, K_DR_: Dubinin–Radushkevich isotherm constant related to adsorption energy, K_D_: Hill constant, K_E_: Elovich equilibrium constant, K_F_: Freundlich isotherm constant, K_J_: Jovanovich constant, K_L_: Langmuir equilibrium constant, K_RP_: Redlich–Peterson isotherm constant, K_T_: Temkin isotherm equilibrium binding constant, n_F_: Freundlich constant, n_H_: Hill cooperativity coefficient of the binding interaction, n_K_: Koble–Corrigan model exponent, q_DR_: Dubinin–Radushkevich theoretical isotherm saturation capacity, q_e_: equilibrium adsorption capacity of adsorbent, q_mE_: Elovich maximum adsorption capacity, q_mJ_: Jovanovich maximum adsorption capacity, q_mL_: Langmuir maximum adsorption capacity of adsorbent, q_mS_: Sips maximum adsorption capacity, q_mT_: Toth maximum adsorption capacity, q_SH_: Hill theoretical isotherm saturation capacity, q_SK_: Khan theoretical isotherm saturation capacity, R: universal gas constant (8.314 J/mol K), T: temperature (K), z: Toth model exponent, ε: Polanyi potential constant related to Dubinin–Radushkevich model (ε = RTln(1 + 1/C_e_)].

**Table 2:** Kinetic model fitting parameters for BPA adsorption onto β-CDNS.

Kinetics	Equation	Model Parameters			$R^{2}$
Pseudo–first order	$q_{t}=q_{e}\left(1-e^{-k_{1}t}\right)$	$q_{e}$	$k_{1}$		0.9739
		46.9502	0.0781		
Pseudo–second order	$q_{t}=\frac{q_{e}^{2}k_{2}t}{1+q_{e}t}$	$q_{e}$	$k_{2}$		0.9977
		48.3900	0.0031		
Mixed order	$\frac{{dq}_{t}}{dt}=k_{1}^{\prime }\left(q_{e}-q_{t}\right)+k_{2}^{\prime }{\left(q_{e}-q_{t}\right)}^{2}$	$q_{e}$	$k_{1}^{\prime }$	$k_{2}^{\prime }$	0.9996
		47.7050	0.0119	0.0026	
Pseudo–nth order model	$q_{t}=q_{e}-{\left(\left(n-1\right)k_{n}t+q_{e}^{1-n}\right)}^{\frac{1}{1-n}}$	$q_{e}$	$k_{n}$	n	0.9991
		47.8647	0.0074	1.7269	
Elovich	$q_{t}=\frac{1}{\beta }ln\left(1+\alpha \beta t\right){\left(q_{e}-q_{t}\right)}^{2}$	$q_{e}$	α	β	0.8429
		49.1931	3.0549 × 10^5^	0.3790	
Avrami	$q_{t}=q_{e}\left(1-e^{-k_{A}t^{n_{A}}}\right)$	$q_{e}$	$k_{A}$	$n_{A}$	0.9997
		47.5724	0.2155	0.6078	

[$q_{t}$: adsorption capacity at time t, $q_{e}$: equilibrium adsorption capacity, $k_{1}$, $k_{2}$, ${k\prime }_{1}$, ${k\prime }_{2}$, $k_{n}$, $k_{A}$: kinetic rate constants, α: initial adsorption rate, β: desorption parameter, n: reaction order (pseudo–nth order model), n_A_: Avrami exponent, t: time].

**Table 3:** Thermodynamic parameters of Bisphenol A adsorption.

T (°C)	ΔG^o^ (J/mol)	ΔH^o^ (kJ/mol)	ΔS^o^ (J/mol K)
15	−5130.5	−11.723	−23.035
25	−4853.8		
35	−4539.9		
45	−4476.7		

## Data Availability

The original contributions presented in this study are included in the article. Further inquiries can be directed to the corresponding author.
